# Clinical burden of severe respiratory syncytial virus infection during the first 2 years of life in children born between 2000 and 2011 in Scotland

**DOI:** 10.1007/s00431-019-03564-9

**Published:** 2020-01-07

**Authors:** Richard Thwaites, Scot Buchan, John Fullarton, Carole Morris, ElizaBeth Grubb, Barry Rodgers-Gray, Jonathan Coutts

**Affiliations:** 1 Portsmouth Hospitals NHS Trust, Queen Alexandra Hospital, Portsmouth, PO6 3LY UK; 2 Strategen Limited, Basingstoke, UK; 3 Information Services Division Scotland, Farr Institute Scotland, Edinburgh, UK; 4 Health Economics & Outcomes Research, AbbVie Inc, North Chicago, IL USA; 5 Royal Hospital for Children, Glasgow, UK

**Keywords:** Respiratory syncytial virus (RSV), Lower respiratory tract infection (LRTI), RSV hospitalisation, Bronchiolitis

## Abstract

National data from Scotland (all births from 2000 to 2011) were used to estimate the burden associated with respiratory syncytial virus hospitalisation (RSVH) during the first 2 years of life. RSVHs were identified using the International Classification of Diseases 10th Revision codes. Of 623,770 children, 13,362 (2.1%) had ≥ 1 RSVH by 2 years, with the overall rate being 27.2/1000 (16,946 total RSVHs). Median age at first RSVH was 137 days (interquartile range [IQR] 62–264), with 84.3% of admissions occurring by 1 year. Median length of stay was 2 (IQR 1–4) days and intensive care unit (ICU) admission was required by 4.3% (727) for a median 5 (IQR 2–8) days. RSVHs accounted for 6.9% (5089/73,525) of ICU bed days and 6.2% (64,395/1,033,121) of overall bed days (5370/year). RSVHs represented 8.5% (14,243/168,205) of all admissions between October and March and 14.2% (8470/59,535) between December and January. RSVH incidence ranged from 1.7 to 2.5%/year over the study period. Preterms (RSVH incidence 5.2%), and those with congenital heart disease (10.5%), congenital lung disease (11.2%), Down syndrome (14.8%), cerebral palsy (15.5%), cystic fibrosis (12.6%), and neuromuscular disorders (17.0%) were at increased risk of RSVH.

*Conclusions*: RSV causes a substantial burden on Scottish paediatric services during the winter months.

**Table:** 

**What is known:** • *Respiratory syncytial virus (RSV) is a leading cause of childhood hospitalisation.*	
**What is new:** • *This 12-year study is the first to estimate the burden of RSV hospitalisation (RSVH) in Scotland and included all live births from 2000 to 2011 and followed > 600,000 children until 2 years old.* *• The overall RSVH rate was 27.2/1000 children, with 2.1% being hospitalised ≥ 1 times.* *• RSVHs accounted for 6.2% of all inpatient bed days, which rose to 14.2% during the peak months of the RSV season (December–January), equating to over 1400 hospitalisations and nearly 5500 bed days each year.*

**What is known:**

• *Respiratory syncytial virus (RSV) is a leading cause of childhood hospitalisation.*

**What is new:**

• *This 12-year study is the first to estimate the burden of RSV hospitalisation (RSVH) in Scotland and included all live births from 2000 to 2011 and followed > 600,000 children until 2 years old.*

*• The overall RSVH rate was 27.2/1000 children, with 2.1% being hospitalised ≥ 1 times.*

*• RSVHs accounted for 6.2% of all inpatient bed days, which rose to 14.2% during the peak months of the RSV season (December–January), equating to over 1400 hospitalisations and nearly 5500 bed days each year.*

## Introduction

Respiratory syncytial virus (RSV) is a major cause of lower respiratory tract infections (LRTI) in early childhood, resulting in a substantial burden on health care services, particularly during the colder months (typically October–March) in Northern Hemisphere countries [1–5]. Robust epidemiological data on RSV LRTI aids planning, guides preventive strategies and helps manage healthcare resources effectively. Quantifying the burden of RSV LRTI at a national level can be difficult, due to the limited availability of suitable data sources and a lack of routine testing and confirmatory diagnoses of RSV. Study methodologies have included utilisation of databases of hospital records or medical insurance [3, 4] or modelling hospital admissions with national surveillance data [5]. Studies from England have reported RSV hospitalisation (RSVH) rates of 2.4–3.5% in children < 1 year [5, 6], rising to 14.3% in those born < 32 weeks’ gestational age [wGA] [7]. To our knowledge, no studies reporting such data from Scotland have been published.

The Information Services Division (ISD) Scotland collates comprehensive national datasets covering all aspects of hospital care within National Health Service (NHS) Scotland, providing ‘linked’ data on the total population (> 5 million people) [8]. The aim of this study was to evaluate the hospital burden associated with RSV during the first 2 years of life in children born within NHS Scotland from 2000 to 2011.

## Materials and methods

### Study population

Data on all live births were extracted from the ISD Scottish Maternity and Birth Record dataset (SMR02) for the period 2000–2011 and each child was followed for 2 years. Those who died during the study period for any reason other than RSV-related infection, those whose records were unable to be linked between datasets (SMR02, Scottish Birth Record [SBR], General/Acute Inpatient and Day Case Record [SMR01], and Outpatient Attendance Record [SMR00]), or those who moved away from Scotland were excluded.

### Demographic factors

The following demographic factors were assessed: sex, gestational age at birth, birthweight, 5-min Apgar score, need for emergency caesarean, multiple births, mother’s age when giving birth, mother’s smoking history at pre-delivery assessment (self-declared) and previous pregnancies. Socio-economic status was determined using the Scottish Index of Multiple Deprivation (SIMD) based on the postcode (ZIP code) of the mother, where 1 = most deprived and 5 = least deprived [9].

### Identification of RSVHs

RSVHs were defined and analysed as having one of the following three International Classification of Diseases 10th Revision (ICD-10) codes: J12.1 (RSV pneumonia); J20.5 (acute bronchitis due to RSV); or J21.0 (acute bronchiolitis due to RSV) (defined as ‘definite’ RSV; Table 1). Whilst RSV testing is routinely undertaken in virtually all paediatric inpatient units in Scotland, to provide an estimate of the maximal impact of RSVH, additional ICD-10 codes were analysed covering ‘probable’ and ‘possible’ RSVHs. The likelihood of the ‘probable’ and ‘possible’ groups being true RSV diagnoses was analysed in two ways. Firstly, these groups were compared to the ‘definite’ group in terms of seasonality. Secondly, any hospitalisations that occurred out of the RSV season in the ‘probable’ and ‘possible’ groups, above the rate in the ‘definite’ group, were reflected across the whole year and assumed not to be true RSVHs.

**Table 1:** Diagnostic codes used to identify RSVHs and comorbidities

Condition	ICD-10 code	ICD-10 code definition
RSV (definite)	J12.1	RSV pneumonia
	J20.5	Acute bronchitis due to RSV
	J21.0	Acute bronchiolitis due to RSV
RSV (probable)	J20.9	Acute bronchitis unspecified
	J21.9	Acute bronchiolitis unspecified
RSV (possible)	J12.8	Viral pneumonia unspecified
	J12.9	Bronchopneumonia unspecified
	J18.0	Lobar pneumonia unspecified
	J18.9	Pneumonia unspecified
	J22	Unspecified acute LRTI
CHD or PH	Q20-Q26	CHD or PH
CLD or BPD	P27.1, Q30-Q34	CLD or BPD
Down syndrome	Q90	Down syndrome
Turner syndrome	Q96	Turner syndrome
Cystic fibrosis	E84	Cystic fibrosis
Cerebral palsy	G80	Cerebral palsy
Neuromuscular disorders	G71	Neuromuscular disorders
Other	D81.9, Q78.0	Combined immunodeficiency, unspecified; osteogenesis imperfecta

*BPD* bronchopulmonary dysplasia, *CHD* congenital heart disease, *CLD* congenital lung disease, *LRTI* lower respiratory tract infection, *PH* pulmonary hypertension, *RSV* respiratory syncytial virus, RSVH: *RSV* hospitalisation

### Hospital burden of RSV

Details of all RSVHs were captured, including number of children admitted and total admissions, average length of stay (LOS), number of bed days and requirement for admission to an intensive care unit (ICU). RSVH rates (per 1000 children), the seasonality of RSV burden (defined as October–March) and the incidence of RSVH in preterms (≤ 36 wGA) and other recognised high-risk groups, including those with congenital heart disease (CHD)/pulmonary hypertension (PH), congenital lung disease (CLD)/bronchopulmonary dysplasia (BPD), Down syndrome, cystic fibrosis, cerebral palsy and neuromuscular disorders (identified using ICD-10 codes; Table 1), were assessed.

### Statistical analysis

Chi-squared tests and *t* tests, as appropriate, were used to assess differences between groups. The Wilson method was used to calculate 95% confidence intervals for incidence rates. Analysis of RSVHs over time was via linear regression and ANOVA. RSVHs by month of birth were compared by chi-squared test. All analyses were performed using StatsDirect version 2.8.0, SPSS for Windows version 15.0, and Microsoft Excel.

## Results

### Study population

In total, 637,502 children were identified of whom 623,770 were included in the study (11,768 émigrés and 1964 non-RSV-related deaths were excluded). Of the 623,770 children, 13,362 (2.1%) had ≥ 1 RSVH during the first 2 years of life. Children hospitalised for RSV were significantly more likely to be male (56.5% vs. 51.0%), part of a multiple birth (5.6% vs. 2.9%), delivered by emergency caesarean (16.6% vs. 15.2%), have a 5-min Apgar score of < 7 (2.6% vs. 1.3%), and have a lower weight (median 3280 g vs. 3420 g) and lower GA (median 39 vs. 40 wGA) at birth than those not hospitalised for RSV (all *p* < 0.0001) (Table 2). The mothers of RSV-hospitalised children were younger (median age: 28 vs. 29 years), more often from the most deprived areas (SIMD 1 [most deprived] 30.7% vs. 25.5%), were current smokers at pre-delivery assessment (34.0% vs. 23.8%), and had ≥ 1 prior pregnancy (75.0% vs. 63.3%) than their counterparts (all *p* < 0.0001).

**Table 2:** Demographics of children and mothers and incidence of RSVH

Demographic factors		RSVH children	Non-RSVH children	Incidence of RSVH (95% CI)
Number of children		13,362	610,408	2.1% (2.1–2.2%)
Mothers				
Age at giving birth (years)	Mean (SD)	28.1 (6.1)	28.9 (6.1)*	–
	Median [IQR]	28 [23–33]	29 [24–33]	–
SIMD Quintile, *n* (%)	1—most deprived	4090 (30.7)	154,969 (25.5)*	2.6% (2.5–2.7%)
	2	2898 (21.7)	125,557 (20.6)	2.3% (2.2–2.3%)
	3	2319 (17.4)	112,694 (18.5)	2.0% (1.9–2.1%)
	4	2073 (15.6)	110,366 (18.1)	1.8% (1.8–1.9%)
	5—least deprived	1947 (14.6)	105,278 (17.3)	1.8% (1.7–1.9%)
Smoking history at pre-delivery assessment, *n* (%)	Current	4095 (34.0)	131,157 (23.8)*	3.0% (2.9–3.1%)
	Former	1141 (9.5)	58,859 (10.7)	1.9% (1.8–2.0%)
	Never	6805 (56.5)	360,410 (65.5)	1.9% (1.8–1.9%)
Previous pregnancies, *n* (%)	0	3336 (25.0)	223,800 (36.7)	1.5% (1.4–1.5%)
	1	4346 (32.5)	188,201 (30.8)^†^	2.3% (2.2–2.3%)
	2+	5680 (42.5)	198,404 (32.5)^†^	2.8% (2.7–2.9%)
Children and births				
Gender, *n* (%)	Male	7554 (56.5)	311,535 (51.0)*	2.4% (2.3–2.4%)
Multiple births, *n* (%)	Singleton	12,616 (94.4)	592,912 (97.1)	2.1% (2.0–2.1%)
	Multiple	744 (5.6)	17,469 (2.9)*	4.1% (3.8–4.4%)
Emergency caesarean, *n* (%)	Percentage requiring	2224 (16.6)	92,738 (15.2)*	2.3% (2.2–2.4%)
Apgar score, *n* (%)	< 7	347 (2.6)	8022 (1.3)*	4.1% (3.7–4.6%)
	≥ 7	12,795 (97.4)	593,129 (98.7)	2.1% (2.1–2.1%)
Gestational age at birth (weeks)	Mean (SD)	38.2 (2.9)	39.3 (2.1)*	–
	Median [IQR]	39 [38–40]	40 [39–41]	–
Birthweight (g)	Mean (SD)	3177 (761)	3395 (586)*	–
	Median [IQR]	3280 [2810-3680]	3420 [3070–3770]	–

*CI* confidence interval, *IQR*: interquartile range, *RSVH* respiratory syncytial virus hospitalisation, *SD* standard deviation, *SIMD* Scottish Index of Multiple Deprivation

*p < 0.0001 vs. RSVH group; ^†^*p* < 0.0001 for combined ≥ 1 pregnancies vs. RSVH group

### Incidence and burden of RSVH

**Fig. 1:**
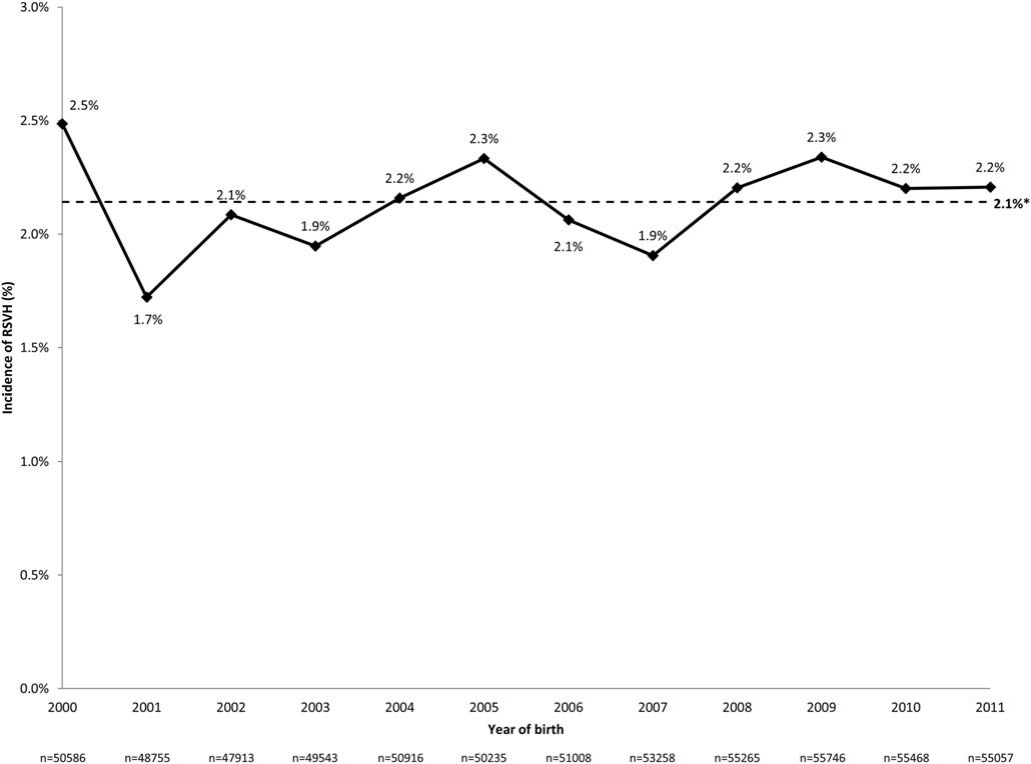
Incidence of RSVHs by year of birth. *Weighted mean; significance of non-zero linear relationship/trend over time: ANOVA, *p* = 0.20

**Fig. 2:**
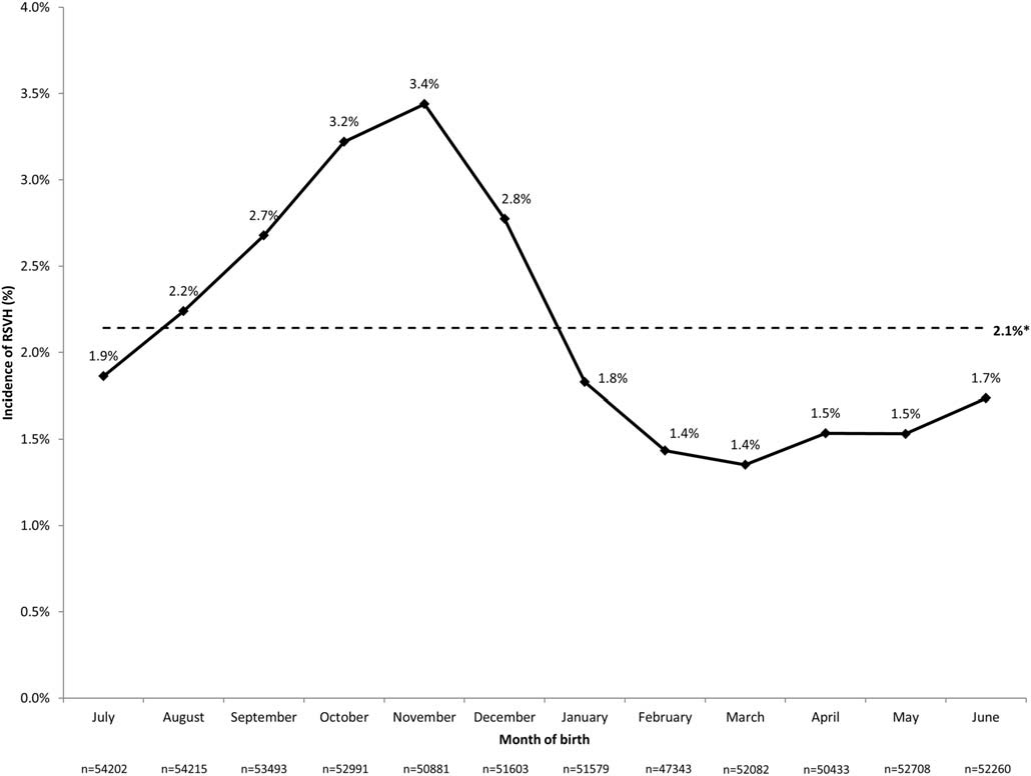
RSVH incidence by month of birth. *Weighted mean

The RSVH rate was 27.2/1000 children (16,946 total RSVHs), equating to 1410 RSV admissions per year over the study period. Readmissions for RSV occurred in 19.1% (2547) of children. The median age at first RSVH was 137 days (interquartile range [IQR] 62–264), with 84.3% of admissions occurring in the first year of life. Median inpatient LOS was 2 (IQR 1–4) days (mean 3.8 days, standard deviation [SD] 13.1). ICU admission was required for 4.3% (727) of cases for a median of 5 (IQR 2–8) days (mean 7.0 days, SD 10.0). RSVHs accounted for 6.9% (5089/73,525) of ICU bed days and 6.2% (64,395/1,033,121) of overall bed days (5370 per year) for children ≤ 2 years during the study period. The majority (91.7%; 15,538) of RSVHs occurred between October and March, representing 8.5% (14,243/168,205) of all admissions in children ≤ 2 years during this time period. During the peak months of December and January, RSV accounted for 14.2% (8470/59,535) of all such admissions. RSVH rates varied across years and RSV seasons, ranging from 1.7% of children born in 2001 (840/48,775) to 2.5% (1258/50,586) in 2000, with no significant (*p* = 0.20) change in incidence seen over the study period (Fig. 1). The RSVH rate also varied by month of birth, with a rate higher than the study mean (2.1%) observed between August and December (range 2.2–3.4%; Fig. 2). The RSVH rate was highest for children born in October (3.2%) and November (3.4%), when it was over 2-fold higher than the rate for children born in March (month with lowest RSVH rate: 1.4%; both *p* < 0.001 vs. March).

### RSVH in children born prematurely and those with comorbidities

Preterms (≤ 36 wGA) were at a significantly increased risk of RSVH compared to children born at term (5.2% vs. 1.9%, respectively; *p* < 0.0001) (Table 3). The risk of RSVH decreased with increasing GA, but remained significantly higher versus term for all groups analysed: < 29 wGA 12.8%; 29–32 wGA 8.2%; 33–35 wGA 4.6% (all *p* < 0.0001). The RSVH rate was significantly higher in children from multiple births than singletons (40.6 vs. 20.8/1000, respectively; *p* < 0.0001), with this being most pronounced in preterm (≤ 35 wGA) than term children (64.8 vs. 23.2/1000; *p* < 0.0001).

**Table 3:** Incidence of RSVH in preterms and other high-risk groups

		RSVH children	Non-RSVH children	Incidence of RSVH (95% CI)
Gestational age at birth	< 29 weeks	277	1891	12.8%* (11.4–14.3%)
	< 32 weeks	839	5453	13.3%* (12.5–14.2%)
	29–32 weeks	562	6329	8.2%* (7.5–8.8%)
	< 36 weeks	1710	26,279	6.1%* (5.8–6.4%)
	≤ 36 weeks	2320	42,319	5.2%* (5.0–5.4%)
	33–35 weeks	871	18,059	4.6%* (4.3–4.9%)
	> 36 weeks	11,023	567,711	1.9% (1.9–1.9%)
Comorbidities	CHD and PH	534	4576	10.5%^‡^ (9.6–11.3%)
	CLD and BPD	228	1803	11.2%^‡^ (9.9–12.7%)
	Down syndrome	87	500	14.8%^‡^ (12.0–18.0%)
	Turner syndrome	1	42	2.3% (0.1–12.3%)
	Cystic fibrosis	30	209	12.6%^‡^ (8.6–17.4%)
	Cerebral palsy	61	332	15.5%^‡^ (12.1–19.5%)
	Neuromuscular disorders	8	39	17.0%^‡^ (7.6–30.8%)
	Other^†^	2	41	4.7% (0.6–15.8%)
No comorbidities		12,411	602,866	2.0% (2.0–2.1%)

*BPD* bronchopulmonary dysplasia, *CHD* congenital heart disease, *CI* confidence interval, *CLD* congenital lung disease, *PH* pulmonary hypertension, *RSVH* respiratory syncytial virus hospitalisation

*p < 0.0001 vs. term (> 36 weeks’ gestational age); ^‡^*p* < 0.0001 vs. no comorbidities; ^†^congenital immunodeficiency disorders and osteogenesis imperfecta

Relevant comorbidities were diagnosed in 7.1% (951/13,362) of children hospitalised for RSV compared to 1.2% (7542/610,408; *p* < 0.0001) of those not RSV-hospitalised (Table 3). An increased risk of RSVH was seen in children with CHD/PH (10.5%), CLD/BPD (11.2%), Down syndrome (14.8%), cerebral palsy (15.5%), cystic fibrosis (12.6%) and neuromuscular disorders (17.0%), compared with children without a comorbidity (2.0%; all *p* < 0.0001).

### Maximal burden of RSVH

**Fig. 3:**
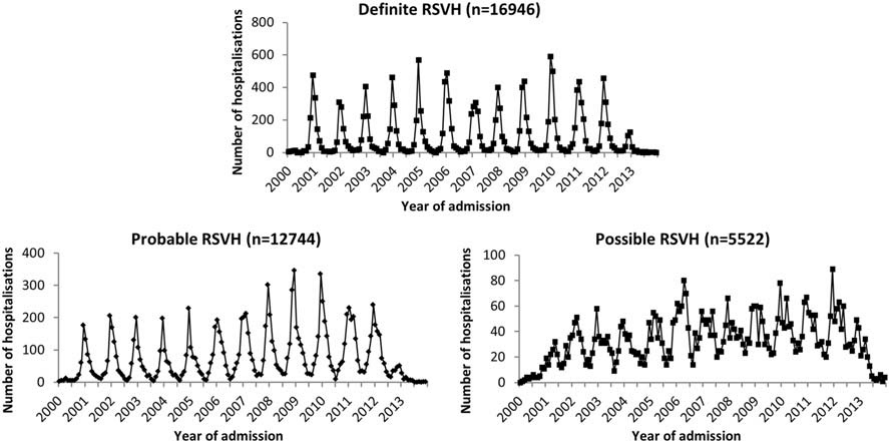
RSVH over time and by certainty of diagnosis. See Table 1 for full group definitions

There were 10,404 children with a ‘probable’ RSVH and 4977 with a ‘possible’ RSVH identified. Combining the ‘definite’, ‘probable’ and ‘possible’ groups gave a maximum of 35,212 admissions for RSV (rate of 56.5/1000 children). These admissions accounted for 10.3% (106,400/1,033,121) of overall bed days for children ≤ 2 years during the study period, and 23.9% (14,257/59,535) of all admissions in December and January. A similar pattern of seasonality was shown by the ‘definite’ and ‘probable’ groups, with 91.7% (15,538/16,946) and 79.8% (10,174/12,744) of hospitalisations occurring during the RSV season; such seasonality was less apparent for the ‘possible’ group (59.6%; 3290/5522) (Fig. 3). By removing excess admissions throughout the year, it was calculated that 23.7% of ‘probable’ and 64.2% of ‘possible’ hospitalisations are likely not be RSV-related. Assuming this to be the case, the maximal RSVH rate was estimated to be 45.9/1000.

## Discussion

This 12-year study of > 600,000 children provides comprehensive evidence of the substantial clinical burden of RSV in children ≤ 2 years of age in Scotland. RSVHs accounted for 6.2% of all inpatient bed days and 6.9% of all ICU bed days in these children. During the peak months of the RSV season, December and January, this increased to approximately one in seven (14.2%) of all bed days. In total, this represented over 1400 hospitalisations and nearly 5500 bed days each year. The overall RSVH rate was 27.2/1000 children over the first 2 years of life, with 2.1% of children being hospitalised ≥ 1 times.

A number of studies and surveillance data from England indicate a rising incidence of RSVH over the past 15 years [5, 10–12], with one study (1995–2009) reporting relatively stable rates [13]. An analysis of 468,138 bronchiolitis episodes (1979–2011) estimated a hospitalisation rate of 46.1/1000 infants aged < 1 year in 2011, with the incidence increasing by 1.8% per year between 2004 and 2011 [10]. Another recent study (2007–2012) estimated a RSVH rate of 35.1/1000 infants < 1 year, based on 121,968 respiratory admissions, with a general increase in RSVHs over the study period [5]. The authors also noted that by restricting the definition of RSVH to those with a primary diagnosis of upper respiratory tract infection and LRTI, they might have underestimated the true burden of disease [5]. This apparent increase in RSVHs perhaps reflects, in part, rising rates of emergency admissions in children in general within the UK [11], increasing use of pulse oximetry, leading to more infants with hypoxaemia being identified and admitted than on purely clinical grounds [14], and more widespread availability of rapid antigen detection tests for RSV at point-of-care. Our study, in contrast, observed a relatively stable rate of RSVH over time in Scotland. This may relate to RSV testing being routine in Scotland over the study period (email communication between Scottish paediatric inpatient units and Jonathan Coutts, December 2018).

Differing methodologies, data sources, populations and RSVH definitions complicate comparisons between studies. In general, earlier studies (1990s to early 2000s) have tended to report lower RSVH rates than those conducted in the last decade [5, 6, 13, 15–18]. One study of 15,116 children < 2 years reported a RSVH rate of 16.3/1000 between 1996 and 1999, based on a positive immunofluorescence test [6]. This is just over half the rate found in our study (27.2/1000), albeit this rate was calculated over 2 years so it is not directly comparable. The average LOS (ours: median 2 days vs. median 2 days [6]) and admission to ICU (4.3% vs. 2.7% [6]) in both studies, however, were broadly similar.

Our study provides further evidence for several high-risk groups and known risk factors for RSVH, including birth in proximity to the RSV season, maternal smoking, low social class/deprivation, male sex, siblings in the household, and multiple births [19–21]. Children born prematurely were found to be at increased risk of RSVH, including those born moderate-late preterm (33–35 wGA), where the incidence was more than double that in term children (4.6% vs. 1.9%, respectively; *p* < 0.0001). These data support the case for preventive strategies against severe RSV infection in preterm infants [22–24]. In addition to established high-risk comorbidities, such as CLD/BPD and CHD, our study provides valuable new data on the increased risk of RSVH in children with Down syndrome (RSVH incidence: 14.8%), cystic fibrosis (12.6%), cerebral palsy (15.5%), and neuromuscular disorders (17.0%), where published data are more scarce [25].

The primary analysis in this paper focussed on the RSV diagnoses J12.1, J20.5 and J21.0 and, with RSV testing routine in almost all paediatric inpatient units in Scotland, this is likely to provide a robust estimate of the burden of RSVH in Scotland. However, it is acknowledged that this approach has certain limitations in that it relies on coding precision and might, therefore, underestimate the true burden of RSVH. By including additional ICD-10 codes for ‘probable’ and ‘possible’ RSV cases, as has been done in other studies [2, 5], it was estimated that the maximal rate of RSVH was 45.9/1000. Another limitation is that there is no method to identify through the ISD databases those children that received palivizumab prophylaxis, although during the time period palivizumab use was restricted on a case-by-case basis to infants < 12 months with extreme prematurity, acyanotic CHD, congenital or acquired significant lung diseases, and immunodeficiency [26]; hence, the impact on overall rates would have been minimal.

This study, which covered all live births from 2000 to 2011, provides a reliable estimate of the significant and rising burden of RSVH in young children (< 2 years) in Scotland. These data can aid the planning and delivery of preventive strategies and care and highlight the need for the development of an effective vaccine.

## Abbreviations

BPD: Bronchopulmonary dysplasia
CHD: Congenital heart disease
CLD: Congenital lung disease
ICD 10: International Classification of Diseases 10th Revision
ICU: Intensive care unit
IQR: Interquartile range
ISD: Information Services Division
LOS: Length of stay
LRTI: Lower respiratory tract infection
NHS: National Health Service
PH: Pulmonary hypertension
RSV: Respiratory syncytial virus
RSVH: Respiratory syncytial virus hospitalisation
SD: Standard deviation
SIMD: Scottish Index of Multiple Deprivation
wGA: Weeks’ gestational age
